# Silent onset of postmenopausal endometriosis in a woman with renal failure in hormone replacement therapy: a case report

**DOI:** 10.1186/1752-1947-4-248

**Published:** 2010-08-04

**Authors:** Ugo Indraccolo, Fabrizio Barbieri

**Affiliations:** 1 Maternal-Child Department, Operative Unit of Obstetrics and Gynecology, ULSS 17 - Veneto, Monselice (PD), Italy; 2 Department of Surgical Sciences, Institute of Obstetrics and Gynecology, University of Foggia, Foggia, Italy; 3 Via Montagnano 16, 62032 Camerino (MC), Italy.Tel: 39 328 6180677, Fax: 39 0737 636668; 4 Dipartimento materno infantile, U.O. di Ostetricia e Ginecologia, Ospedale di Monselice, Via Marconi 19, 35043 Monselice (PD), Italy

## Abstract

**Introduction:**

Postmenopausal endometriosis is a rare form of a common disease, since the absence of estrogenic hormone production should halt disease progression.

**Case presentation:**

We present the case of a 54-year-old Italian Caucasian woman in surgical menopause with a history of ovarian endometriosis, who underwent voluntary hormone replacement therapy for seven years. She developed postrenal renal failure due to bilateral compression of the pelvic ureteral tract caused by two large, deeply infiltrating endometriotic nodules with no pelvic pain. She underwent operative laparoscopy with adhesiolysis of enteroenteric adhesions and excision of the endometriotic nodules encompassing the juxtavesical tract of the ureters, without obtaining improvement of renal failure.

**Conclusion:**

Postmenopausal endometriosis can manifest itself in an unpredictable and potentially very serious manner. It is therefore important to carefully evaluate the risks and benefits of administering hormone replacement therapy to patients with previous endometriosis.

## Introduction

Postmenopausal endometriosis is a rare form of a common disease, given that the absence of estrogenic hormone production should halt disease progression [1]. Oxholm *et al *[2] reported that two to five percent of endometriosis is diagnosed after menopause. It has been reported that endometriosis may develop essentially in women undergoing hormone replacement therapy [2] with some exceptions [3], indicating the possibility that in some cases endometriosis may be completely independent of gonadic estrogens. Whether postmenopausal endometriosis is due to exogenous estrogens or presumably independent of gonadic estrogens, the silent growth of the disease can result in potentially serious and unpredictable complications. For example, it may grow without the typical symptoms such as catamenial pain and may involve the ureters [4,5] or bowel [6], producing complications such as renal failure or intestinal obstruction. The following case explains the onset of postmenopausal endometriosis with renal failure.

## Case presentation

A 54-year-old Italian Caucasian woman, weighing 71 kg and with a height of 160 cm, was admitted to our facility in order to have a laparoscopic removal of two nodules compressing both ureters. She had received diagnosis of endometriosis laparoscopically, when she was 43. At 44 years of age, she underwent a total laparotomic hysterectomy with bilateral adnexectomy for metrorrhagia from uterine fibromatosis. During the operation and after pathological examination, no sign of endometriosis was found. Subsequently, she underwent voluntary hormone replacement therapy (estrogen-based only) for seven years with good general health until the detection, during the eighth year of menopause, of renal failure due to bilateral hydronephrosis (detected via MRI). The bilateral hydronephrosis was induced by extrinsic compression of both ureters (at supravesical fossa) by nodules compatible with deeply infiltrating endometriosis. S-Ca 125 appeared within the norm (normal values are considered below 31 microU/ml) and no pelvic pain was reported. Upon hospitalization, five months after instrumental diagnosis and following subsequent ureteral stenting, her creatinine value was 1.71 mg/dl (range 0.66 to 1.09 mg/dl), with blood urea nitrogen at 57 mg/dl (range 17 to 43 mg/dl). S-Ca 125 again appeared to be within the normal range (below 31 microU/ml). She then underwent operative laparoscopy with adhesiolysis of entero-enteric adhesions and excision of endometriotic nodules encompassing the juxtavesical tract of the ureters: on the right extending to the external iliac artery and obturator foramen and on the left, to the rectum. Pathological examination of the excised nodules confirmed the instrumental and laparoscopic diagnosis of postmenopausal endometriosis. Post-operative recovery was complicated by broncho-pneumonia. After hospital discharge, creatinine value was 1.56 mg/dl (range 0.66 to 1.09 mg/dl) with thinning of the right renal cortex which suggested mild renal failure. Following removal of the ureteral stents three months after surgery, the patient appeared to be in good health despite the mild renal failure.

## Discussion

For more than 10 years it has been acknowledged [7,8] that endometriosis can express aromatase activity, particularly during tissue inflammation. In a recent review, Attar and Bulun [9] illustrate how endometriosis can express various enzymes from the biosynthetic pathway of steroid hormones: estrogen production is caused by aromatase activity during inflammatory episodes; in addition, estrogen production can increase inflammation of endometriotic tissue. During menopause it is plausible that endometriosis can grow independently of gonadic estrogens due to the renewed synthesis of estrogens in the endometriotic nodule. Therefore, the effect of estrogens may be variable overall, because inflammation affects estrogen production in the nodule. In addition, Rosa-e-Silva *et al *[3] proposed that obesity may have a particular role in the growth of post-menopausal endometriosis due to estrogen production by fatty tissue. Oxholm *et al *[2] have recently reviewed cases of postmenopausal endometriosis, pointing out that the majority of postmenopausal endometriosis is detected in patients undergoing hormone replacement therapy, particularly when only estrogen-based. In addition, the onset of endometriosis during menopause appears to be more probable following physiological menopause, suggesting that the ovaries may have a certain role in the disease even in the post-menopausal phase. The authors [2] conclude that it is debatable overall whether hormone replacement therapy can favor the growth of endometriosis, implying, however, that this is possible in some cases and that endometriosis should be taken into consideration in menopausal patients presenting the pain symptoms typical of the disease.

## Conclusion

Even in fertile patients, endometriosis shows varying biological behavior, with variable clinical symptoms and outcomes in relation to hormonal status. However, it is very difficult for a clinician to ascertain silent endometriosis post-menopause, when estrogen production is lacking. In light of this case and of the varying biological behavior of endometriosis, clinicians must certainly keep in mind that postmenopausal endometriosis can appear in an atypical manner and could go undetected, leading to serious complications. This event may occur particularly in patients with a type of hormonal trigger that could aggravate inflammatory stress. Therefore, we recommend a careful evaluation of whether or not to prescribe hormone replacement therapy to patients in menopause with previously ascertained endometriosis.

## Patient perspective

The patient was not happy about her experience and considers endometriosis a painful disease that she had hoped would be cured with her hysterectomy with adnexectomy. She hopes that the description of her experience will be helpful in preventing and treating the troubles that such a disease can provoke.

## Abbreviations

dl: deciliter; mg: milligram; microU: micro-unit; ml: milliliter; MRI: magnetic resonance imaging; S-Ca 125: serum Ca-125

## Competing interests

The authors declare that they have no competing interests.

## Authors' contributions

UI collected the bibliography, read it and was the major contributor in writing the article. FB performed laparoscopic debulking of deep infiltrating endometriosis. Both authors have read and approved the final manuscript.

## Consent

Written informed consent was obtained from the patient for publication of this case report. A copy of the written consent is available for review by the Editor-in-Chief of this journal.
